# Supermarket/Hypermarket Opportunistic Screening for Atrial Fibrillation (SHOPS-AF): A Mixed Methods Feasibility Study Protocol

**DOI:** 10.3390/jpm12040578

**Published:** 2022-04-04

**Authors:** Ian D. Jones, Deirdre A. Lane, Robyn R. Lotto, David Oxborough, Lis Neubeck, Peter E. Penson, Gabriela Czanner, Andy Shaw, Emma Johnston Smith, Aimeris Santos, Emily E. McGinn, Aderonke Ajiboye, Nicola Town, Gregory Y. H. Lip

**Affiliations:** 1 School of Nursing and Allied Health, Faculty of Health, Liverpool John Moores University, Liverpool L3 2AJ, UK; r.r.lotto@ljmu.ac.uk (R.R.L.); e.crawford@ljmu.ac.uk (E.J.S.); aimeris20@hotmail.com (A.S.); emcginn@hotmail.co.uk (E.E.M.); aderonke_ajiboye@yahoo.com (A.A.); n.j.town@ljmu.ac.uk (N.T.); 2 Liverpool Centre for Cardiovascular Science, Liverpool John Moores University, Liverpool L3 2AJ, UK; deirdre.lane@liverpool.ac.uk (D.A.L.); d.l.oxborough@ljmu.ac.uk (D.O.); p.penson@ljmu.ac.uk (P.E.P.); g.czanner@ljmu.ac.uk (G.C.); gregory.lip@liverpool.ac.uk (G.Y.H.L.); 3 Liverpool Centre for Cardiovascular Science, University of Liverpool and Liverpool Heart & Chest Hospital, Liverpool L7 8TX, UK; 4 Department of Clinical Medicine, Aalborg University, 9000 Aalborg, Denmark; 5 School of Sport and Exercise Science, Liverpool John Moores University, Liverpool L3 3AF, UK; 6 School of Health and Social Care, Edinburgh Napier University, Edinburgh EH11 4DN, UK; l.neubeck@napier.ac.uk; 7 School of Pharmacy and Biomolecular Sciences, Liverpool John Moores University, Liverpool L3 3AF, UK; 8 School of Computer Science and Mathematics, Liverpool John Moores University, Liverpool L3 3AF, UK; 9 School of Civil Engineering and Built Environment, Liverpool John Moores University, Liverpool L3 3AF, UK; a.shaw@ljmu.ac.uk

**Keywords:** atrial fibrillation detection, cardiac arrhythmia, stroke prevention, electrocardiogram, sensor devices, community screening

## Abstract

Aims: Atrial fibrillation (AF) is the most common sustained cardiac arrhythmia and a key risk factor for ischaemic stroke. Following AF detection, treatment with oral anticoagulation can significantly lower mortality and morbidity rates associated with this risk. The availability of several hand-held devices which can detect AF may enable trained health professionals to adopt AF screening approaches which do not interfere with people’s daily routines. This study aims to investigate the effectiveness of a hand-held device (the MyDiagnostick single-lead Electrocardiogram (ECG) sensor) in screening for AF when embedded into the handles of supermarket trolleys. Methods: A mixed methods two-phase approach will be taken. The quantitative first phase will involve the recruitment of 2000 participants from a convenience sample at four large supermarkets with pharmacies. Prospective participants will be asked to conduct their shopping using a trolley embedded with a MyDiagnostick sensor. If the device identifies a participant with AF, the in-store pharmacist will be dispatched to take a manual pulse measurement and a static control sensor reading and offer a cardiologist consultation referral. When the sensor does not detect AF, a researcher will confirm the reading with a manual pulse measurement. ECGs will be compiled, and the sensitivity, specificity and positive and negative predictive values will be determined. A qualitative second phase will consist of semi-structured interviews carried out with those pharmacists and store managers in-store during the running of the trial period. These will explore the perceptions of staff regarding the merits of embedding sensors in the handles of supermarket trolleys to detect AF. Conclusion: This feasibility study will inform a larger future definitive trial.

## 1. Introduction 

Cardiovascular disease accounts for almost a quarter (24.7%) of all premature deaths in men and nearly a fifth (17%) in women in the U.K. [1]. Four percent of these deaths are stroke-related [1]. Indeed, while stroke mortality rates have reduced over time, the condition remains one of the major global causes of mortality and disability [2]. In the U.K., stroke is the third most common cause of years of life lost and of disability [3] and is estimated to cost the UK Health and Social Care system GBP 8.6 billion per annum [4]. This cost equates to GBP 22,429 in the first year, with ongoing five-year costs of GBP 46,039 per patient [2]. Eighty-seven percent of these admissions (126,640) are ischaemic stroke-related, and atrial fibrillation (AF) is responsible for around a quarter of such strokes, which are more likely to be severe and fatal compared to other causes of stroke [5,6]. Unlike many other cardiac conditions, recent UK registry-based data has noted that the prevalence of AF is increasing [1], which, if not treated effectively, will likely result in an increasing number of strokes in the longer term, with a major impact on NHS healthcare costs related to AF [7]. Moreover, Public Health England estimates that while around 123,000 people in England are living with AF, around 20% of these people are undiagnosed and are therefore untreated [8].

Whilst it is recognised that the risk of stroke in patients with AF is not homogeneous, all patients with AF should be risk-assessed using the CHA_2_DS_2_-VASc score [9,10], with most patients, except the very lowest risk (CHA_2_DS_2_-VASc score of 0 in men and 1 in women), receiving oral anticoagulation therapy. Anticoagulant therapy, using either vitamin K antagonists (VKAs) or non-vitamin K antagonist oral anticoagulants (NOACS), markedly reduces the risks of stroke and mortality in AF patients [11,12]. More recent focus has been placed on the detection and characterisation of AF patients, followed by a holistic approach to AF care, given the improved clinical outcomes with integrated care management [13,14].

The detection and diagnosis of AF requires ECG rhythm documentation showing the typical pattern of irregular RR intervals with an absence of discernible P waves [10]. By accepted convention, an episode lasting at least 30 s is considered diagnostic [10]. However, whilst an ECG provides documentary evidence, the presence of an irregular pulse arouses suspicion of AF and whilst not as specific or as sensitive as the ECG, checking an individual’s pulse is much less costly and requires fewer resources. A pulse check is therefore an easy, low cost means of ruling out or raising suspicion of AF, as an approach to AF screening [15].

However, the regularity of a person’s pulse and the normality of their ECG are usually only confirmed as a result of either opportunistic screening [10] or post-event identification. Silent or undetected AF is not uncommon [16,17], and although opportunistic screening is considered cost-effective in some populations [18], it may be seen as an inconvenience to those not currently experiencing symptoms and may be irrelevant to some socioeconomically deprived groups who do not engage with health professionals until a crisis occurs. Therefore, alternative models of screening that can be delivered by appropriately qualified personnel whilst not detracting people from their daily activities are required. Until recently, such a process has been impractical; however, with the advent of new technology, this may now be possible, as a number of validated hand-held devices are available that allow irregular pulses to be detected.

**Figure 1:**
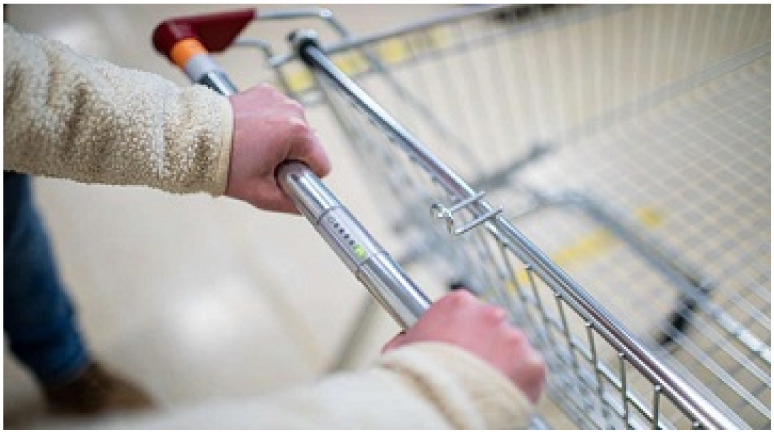
MyDiagnostick single lead ECG sensor embedded into the handles of supermarket trolleys to detect atrial fibrillation. A participant should place both hands around the device for 60 s. Depending on the detection of atrial fibrillation or not, the sensor will present with a red cross or a green tick, respectively.

**Figure 2:**
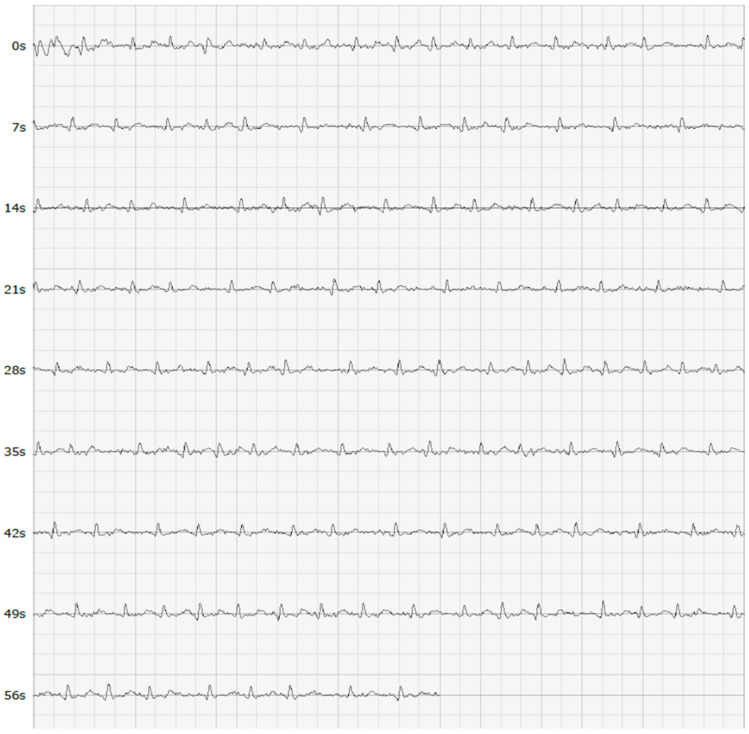
A single lead ECG tracing showing AF.

**Figure 3:**
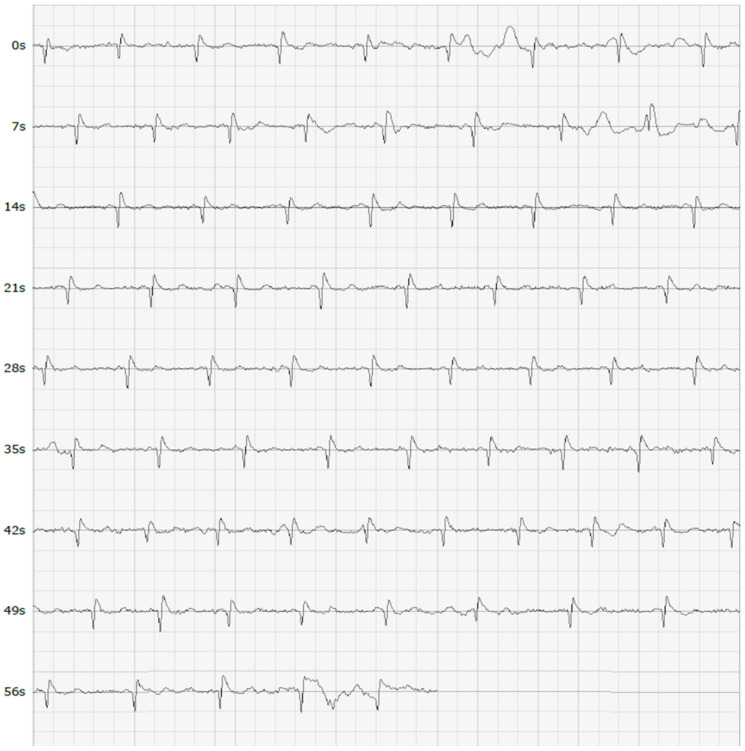
A single lead ECG tracing that was recorded as not AF.

MyDiagnostick is a cylindrical shaped MDD Class IIa medical device (length 26 cm, diameter 2 cm) costing circa GBP 550 that records a single lead I ECG tracing when a participant’s hands make contact with the metal electrodes (Figure 1). The device’s algorithm-based internal software analyses the R-R interval of the ECG recording over a one-minute period whilst contact is maintained. The presence of AF is signified by a red light (Figure 2), whereas the absence of AF is signified by a green light (Figure 3). A single lead tracing can then be downloaded via a USB port onto a computer as a PDF document for further analysis. Whilst the MyDiagnostick algorithm is focussed solely on the presence or absence of AF, the PDF generated allows a trained professional to identify additional arrhythmias if present.

We propose to embed a MyDiagnostick single lead ECG sensor into the handles of supermarket trolleys to assess the person’s heart rhythm whilst they undertake their shopping (Figure 1). If an abnormal heart rhythm is detected, the store’s pharmacist is notified and is dispatched to meet with the shopper. Community-based pharmacies have been identified as a potential location to undertake opportunistic screening and lifestyle interventions [19]. Previous studies have demonstrated that pharmacists are able to successfully screen and identify those at risk of cardiovascular disease [20,21] and is acceptable to patients [22], but again, these studies have relied on self-presentation.

## 2. Methods

We are proposing to locate a research team in four large supermarkets that contain pharmacies where shoppers will be asked to test the embedded technology, and store managers and pharmacists will provide their views on the merits of the screening approach. We will do this using a mixed methods two-phase approach.

### 2.1. Phase 1

#### 2.1.1. Study Design

A cross-sectional observational study with a convenience sample will be used to address the research objectives.

#### 2.1.2. Eligibility Criteria

Members of the public who are visiting four large supermarkets in the North West of England. We will recruit from four supermarkets for a period of two months each. To be eligible for inclusion, participants must be aged ≥18 years, able to grip a shopping trolley handle and provide written informed consent. Participants will be excluded if they have a physical tremor as this affects the ability of the device to record the heart rhythm due to movement artefact and previously participated in the study. Those with known AF will not be excluded from participation as the inclusion of this group of patients will aid the assessment of the sensitivity of the sensor and minimise selection bias.

#### 2.1.3. Sample Size

Large supermarkets attract an average footfall of 25,000 people per week [23], providing a potential total population across four stores per week of 100,000 people. Recognising that many of these people are returning customers attending on a weekly basis, this study aims to recruit around 2% of the total population, resulting in a sample of 2000 participants. The prevalence of AF in the area of the North West of England from which participants are to be recruited is estimated to be 2.1% [24,25], which would result in 42/2000 positive results, including some who are known to experience AF. However, we recognise that those most at risk of developing AF are ≥ 65 years. Unfortunately, neither the British Retail Consortium nor YouGov provides age-related data for in-store supermarket shopping. One American study estimated that those over the age of 60 years make up 24% of supermarket consumers [26]. Recognising that the prevalence of AF is highest in this older age group, ranging from 4–11% in those 65–79 years old, we estimate a total of 480/2000 people will be screened from within this age group and between 19/480 and 53/480 people will be found to be in AF. In addition, it is estimated that the remaining 1520 customers present between 0.1–1.5% risk of AF suggesting between 2/1520 and 23/1520 additional presentations. In total, it is estimated that between 21 and 76 participants will be found to be suffering AF [27]. The prevalence of undiagnosed AF differs dependent on age. A systematic review of 30 single time-point AF screening studies including 122,571 participants (Lowres et al., 2013) reported the incidence of previously unknown AF as 1.0% (CI, 0.89–1.04%), increasing to 1.4% (CI, 1.2–1.6%) in those >65 years old. We recognise that we are recruiting a yonger population than those included in these studies, we therefore estimate that up to 28 people will be identified with undiagnosed AF.

#### 2.1.4. Recruitment

A convenience sample of all those attending four designated supermarkets in the North West of England. We will station a researcher at the entrance of each store who will approach shoppers as they enter the store and invite them to participate in the study. Participant information sheets on the trolley will outline the study and provide details of how people can participate and also include details of the funder (Bristol Myers Squibb). The supermarkets included in the study are located within the Liverpool City Region, a region with pockets of high levels of deprivation. The supermarkets are situated within community hubs and recognise their social responsibility.

#### 2.1.5. Informed Consent

Verbal consent will be gained prior to recruitment, with written consent obtained for all participants with an abnormal sensor reading whose personal data are required for onward referral to a consultant cardiologist.

#### 2.1.6. Sensor Technology

The sensor used within this study, MyDiagnostick has been shown to be highly sensitive in detecting AF, with sensitivity levels ranging between 94% (95% CI 87–98%) [28] to 100% (95% CI 93–100%) [29]. The specificity of MyDiagnostick also compares well with other devices ranging between 93% (95% CI 85–97%) [28] to 95.9% (95% CI 91.3% to 98.1%) [29].

#### 2.1.7. Study Procedures

We will recruit 2000 people attending one of four supermarkets in the North West of England. Each participant will use a supermarket trolley to undertake his or her routine shopping and, by doing so, will grip the trolley handle at differing points and for differing time periods. It will be made clear to each participant that only one person should push the trolley during his or her visit to the store. During each contact with the trolley handle, the sensor will assess the participant’s pulse and store a recording of the rhythm strip, which is stored within the sensor’s file storage system. These data will be downloaded alongside the personal data at the end of each day and deleted from the storage system. The handles of all trolleys will be sanitised between participants to minimise the risk of infection.

**Figure 4:**
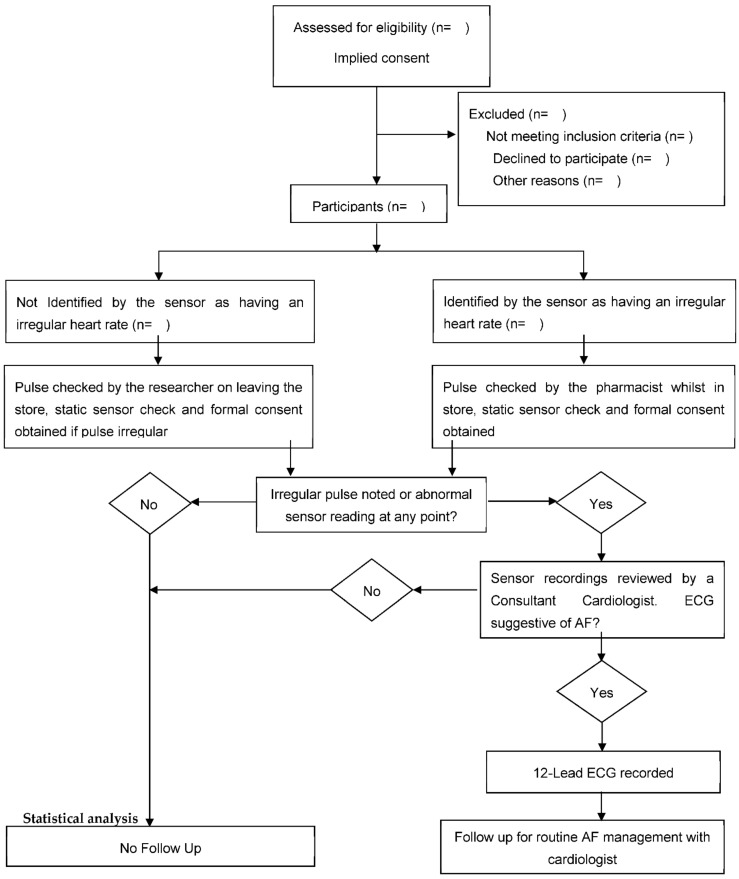
Phase 1 (Quantitative) procedure to follow regarding those participants to be recruited in a cross-sectional observational study determining the effectiveness of sensor technology to detect atrial fibrillation (AF) when embedded in the handles of supermarket trolleys. A minimum of two thousand participants will be recruited. A convenience sample will be used.

If the pulse sensor detects AF, the store pharmacist will be alerted by the researcher, and they will meet with the participant in the store to repeat the sensor check and undertake a manual pulse check. If the pulse sensor does not detect AF whilst the person is pushing the trolley, the researcher will meet with the participant and a manual pulse check will be undertaken once the person has completed their shopping. The procedure to be followed for each participant is outlined in Figure 4. An irregular pulse is defined as any irregularity between pulse waveforms in the radial artery within a period of sixty seconds. Guidance recommends pulse palpation as the first step for AF screening [10]. Two randomised controlled trials have found that pulse palpation is an effective and cost-effective approach for screening for AF [18,30]. All staff undertaking manual pulse checks will undertake additional training using simulation manikins to ensure that they are practising in line with the procedures outlined in the Royal Marsden Manual of Clinical Nursing Procedures [31]. However, a static sensor check (control) will provide an ability to differentiate between normal irregular pulses, e.g., sinus arrhythmia and more harmful conditions, including AF.

#### 2.1.8. Outcome of Interest

The primary objective is to determine the effectiveness of a MyDiagnostick sensor embedded in the handle of a supermarket trolley in detecting AF.

The 12 lead ECGs of all participants referred for follow-up will be analysed for evidence of AF by a Consultant Cardiologist (GL). AF is characterized on the ECG by rapid, irregular waves that vary in size, form and timing. The results of this analysis will be compared with the participant’s initial sensor recording and the static sensor recording to establish concordance.

Within this phase, there are two secondary objectives [1] to establish what percentage of the public will choose to carry out their shopping using a trolley with a pulse sensor in the handle and [2] to establish if shoppers, who have been identified as having an irregular pulse rate, would be willing to attend a local healthcare facility to have an ECG recorded.

The research team will introduce the new trolley to shoppers and the percentage uptake will be identified. These data will provide both the research team and the designers with valuable information that will inform the scalability of the device, if found to be effective.

With consent, details of all those who are found to have an irregular sensor reading will be stored on a research database. A Consultant Cardiologist will review the sensor recordings to eliminate those recordings that are uninterpretable and whereby the sensor has been activated by the presence of the artefact. These participants will be invited to return for an additional static check. The details of those with confirmed AF or suspected AF will be referred to a local healthcare facility. These details will be compared against those who attend a follow-up ECG. Anyone who has not attended an ECG assessment will be contacted by telephone within one week of referral. During this telephone call, the participant will again be made aware of the risks associated with an irregular pulse, and a further invitation to attend a clinic for an ECG will be offered. Participants who do not attend will be contacted on two occasions, after which, if they still have not attended, a letter will be sent to their GP explaining that the detection of an irregular pulse was found during a screening study. Follow-up care will then be provided by the GP as per routine NHS care. All data will be aggregated, enabling comparisons to be made. As the aim of the study is to assess the merit of the screening approach, the study follow-up will discontinue once the participant has attended the cardiology clinic or failed to attend despite two follow-up telephone calls. All treatment beyond this stage will be in line with contemporary NHS AF pathways, and consequently, the likely incidence of stroke will be extrapolated from the results of national datasets.

#### 2.1.9. Statistical Analysis

A sensitivity of 95% and specificity of 90% is sufficiently accurate to be incorporated into clinical practice. If confirmed, they will make supermarkets with in-store pharmacies a nationwide relevant and cost-effective point of screening AF. Such high sensitivity and specificity values are also indicated as plausible by existing published papers, with 95% confidence intervals of 93–100% and 91.3–98.1% [29] and 87–98% and 85–97% [28], for sensitivity and specificity, respectively. In our proposed feasibility study, our precision calculations show that with between 21 and 76 AF cases, we will estimate the 95% confidence interval of sensitivity and specificity with interval widths of 19.0% and 9.8%. The most likely precision will be 12.4%.

Descriptive statistics including simple percentages will be estimated from this feasibility study to demonstrate uptake, rates of detection and successful referral. Generating these data will enable the researchers to determine the number of shoppers who need to be approached to recruit for an adequately powered future study. In addition, conditional percentages will be used to describe the sensitivity and specificity of both the sensor and the pulse check.

By recruiting from community supermarkets based in areas of high deprivation, it is likely that we will recruit a greater number of individuals who are known not to typically engage in traditional screening programmes. This approach attempts to correct the inverse care law, bringing healthcare to the community.

### 2.2. Phase 2

The second phase of the study involves an embedded qualitative approach. The primary objective is to explore the views of supermarket-based pharmacists and store managers on the merits of embedding pulse sensors in the handles of supermarket trolleys to enable the detection of AF. The secondary objective is to explore the pharmacists’ perspectives and experiences of performing manual pulse checks on participants.

#### 2.2.1. Informed Consent

The Senior Store Manager will identify all those staffs who are eligible for the study and will distribute a Participant Information Sheet and Consent Form accordingly. A member of the research team will be available to explain the study to potential participants at a mutually convenient time. All eligible participants will receive information about the study outlining the purpose, potential risks and implications of participation. They will be asked to sign and date an informed consent form that adheres to the ethical principles that have their origin in the Declaration of Helsinki prior to enrolment. The participant information sheet and informed consent form will acknowledge that the study data will be used in academic publications. However, participants will be informed that no personal or identifiable data will be shared outside the research team. All data will be anonymised prior to publication.

#### 2.2.2. Study Design

Qualitative interviews will be undertaken to explore the views of pharmacists and store managers. All pharmacists and store managers from each supermarket will be invited to participate in a qualitative interview. Store managers are defined as any manager with responsibility for managing the store during the study period. Pharmacists are defined as being General Pharmaceutical Council (GPhC) registered and employed in the store. All participants must be able to provide written informed consent and devote time to attend the interview. The exclusion criteria include previous participation in the study in the event of a store manager or pharmacist transferring to a neighbouring store during the study period and absence from the store during the entirety of the data collection period.

#### 2.2.3. Recruitment

A convenience sample including store managers and GPhC registered pharmacists employed within the supermarket within the study period will be recruited. Each store manager will identify potential participants.

#### 2.2.4. Study Procedures

**Figure 5:**
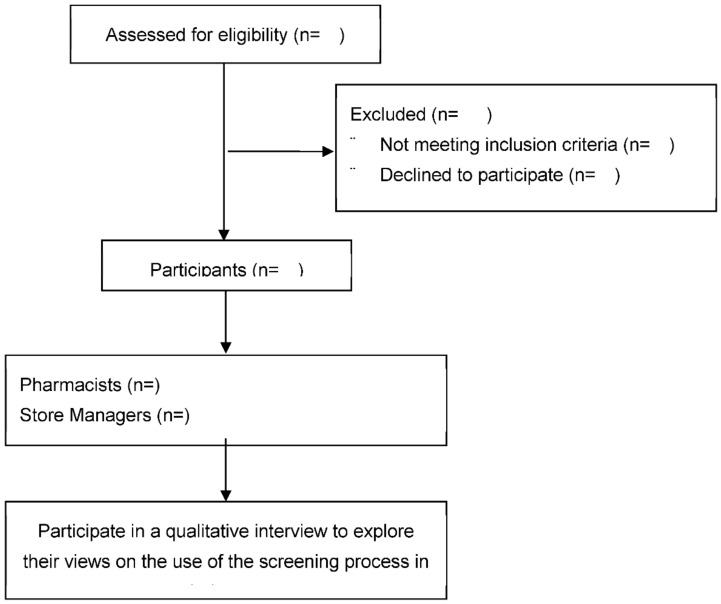
Phase 2 (Qualitative) procedure to follow when conducting semi-structured interviews exploring the views of supermarket-based pharmacists and store managers around the use of sensor technology to detect atrial fibrillation (AF) when embedded in the handles of supermarket trolleys.

We will undertake individual semi-structured interviews with all store managers and pharmacists that have engaged in the screening study. The interviews will focus on exploring the supermarket employees’ views on the merits of embedding sensors in the handles of the supermarket trolleys, the impact of using these trolleys to screen for AF on the workload of the pharmacists and the efficiency of the stores and identify any additional customer feedback that may have been received. The procedure for Phase 2 is outlined in Figure 5.

#### 2.2.5. Data Analysis

Thematic analysis using the qualitative data generated from the interviews with pharmacists and store managers will be independently analysed by two members of the research team adopting Braun and Clarke’s (1996) framework for thematic analysis [32].

#### 2.2.6. Patient and Public Involvement (PPI)

The Service Users for Research Endeavour (SURE) group at Liverpool Heart and Chest Hospital and the patient and public involvement group at the Liverpool Centre for Cardiovascular Science will review the research protocol, participant information sheet and consent form. Their advice will be sought throughout the design of the study.

#### 2.2.7. Ethical Considerations

The ethical review has been granted by Liverpool John Moores University’s University Research Ethics Committee. The study will be undertaken in compliance with the research protocol. During Phase 1, verbal consent will be obtained upon recruitment, with written consent secured for those with an abnormal sensor recording whose personal data will be required for onward referral for 12 lead ECGs. For the qualitative sub-study (Phase 2), written consent will be obtained for all participants.

## 3. Discussion

Many people with AF are undiagnosed, unaware and are subsequently at increased risk of stroke and mortality compared with people with symptomatic AF [33,34,35]. The reasons for these differences are unclear but are likely to occur because of limited preventative treatment, such as anti-coagulation and inadequate management of additional risk factors.

The high economic and individual burden of AF makes early identification through screening an attractive proposition to clinicians, patients and budget holders alike. However, trial data to support AF screening models are scarce [36,37]. Opportunistic screening has been found to be cost-effective [38] but remains reliant on a substantial health resource. Consequently, there is increasing interest in the use of patient-initiated ECG or photoplethysmography screening, with >100 mHealth apps and ≥400 wearable activity monitors reportedly available [39]. However, patient-initiated screening can be expensive to the consumer and consequently excludes those with lower household incomes. Moreover, this form of screening is dependent on individuals with few or no symptoms actively purchasing and participating in a screening ritual. Furthermore, the number of e-health applications is increasing exponentially with little or no scrutiny of their efficacy. It is therefore inevitable that the public will be exposed to poor-quality devices with limited accuracy, the results of which will lead to a combination of false reassurance for some and an inappropriate use of healthcare resources for others. Introducing sensor-based screening into everyday activities with immediate access to resident health care professionals could provide a means of capitalising on the improvements in technology whilst negating the need for individuals to invest in said technology and, in doing so, revolutionise the way that healthcare screening is offered.

This proposed study aims to introduce the concept of using sensor-based diagnostic technology in everyday activities whilst ensuring that all participants have access to a defined healthcare pathway, thus optimising the benefits that early diagnosis provides. The study design will allow the researchers to assess the acceptability of the concept, the efficacy of the sensors and the engagement of those found to be in AF. Finally, the study will address the uncertainties in optimal trial design to inform a future definitive trial.

## References

[1] British Heart Foundation (2018). Cardiovascular Disease Statistics. https://www.bhf.org.uk/what-we-do/our-research/heart-statistics/heart-statistics-publications/cardiovascular-disease-statistics-2018.

[2] Xu X.-M., Vestesson E., Paley L., Desikan A., Wonderling D., Hoffman A., Da Wolfe C., Rudd A.G., Bray B.D. (2018). The economic burden of stroke care in England, Wales and Northern Ireland: Using a national stroke register to estimate and report patient-level health economic outcomes in stroke. Eur. Stroke J..

[3] Newton J.N., Briggs A.D., Murray C.J.L., Dicker D., Foreman K.J., Wang H., Naghavi M., Forouzanfar M.H., Ohno S.L., Barber R.M. (2015). Changes in health in England, with analysis by English regions and areas of deprivation, 1990–2013: A systematic analysis for the Global Burden of Disease Study 2013. Lancet.

[4] Patel A., Berdunov V., Quayyum Z., King D., Knapp M., Wittenberg R. (2020). Estimated societal costs of stroke in the UK based on a discrete event simulation. Age Ageing.

[5] Hannon N., Sheehan O., Kelly L., Marnane M., Merwick A., Moore A., Kyne L., Duggan J., Moroney J., McCormack P.M. (2010). Stroke associated with atrial fibrillation—Incidence and early outcomes in the north Dublin population stroke study. Cerebrovasc. Dis..

[6] Marini C., De Santis F., Sacco S., Russo T., Olivieri L., Totaro R., Carolei A. (2005). Contribution of atrial fibrillation to incidence and outcome of ischemic stroke: Results from a population-based study. Stroke.

[7] Burdett P., Lip G.Y.H. (2020). Atrial Fibrillation in the United Kingdom: Predicting Costs of an Emerging Epidemic Recognising and Forecasting the Cost Drivers of Atrial Fibrillation-related costs. Eur. Heart J. Qual. Care Clin. Outcomes.

[8] NHS digital Quality and Outcomes Framework 2020/2021. https://fingertips.phe.org.uk/search/atrial%20fibrillation.

[9] Lip G.Y., Nieuwlaat R., Pisters R., Lane D.A., Crijns H.J. (2010). Refining clinical risk stratification for predicting stroke and thromboembolism in atrial fibrillation using a novel risk factor-based approach: The euro heart survey on atrial fibrillation. Chest.

[10] Hindricks G., Potpara T., Dagres N., Arbelo E., Bax J.J., Blomström-Lundqvist C., Boriani G., Castella M., Dan G.-A., Dilaveris P.E. (2021). 2020 ESC Guidelines for the diagnosis and management of atrial fibrillation developed in collaboration with the European Association for Cardio-Thoracic Surgery (EACTS): The Task Force for the diagnosis and management of atrial fibrillation of the European Society of Cardiology (ESC) Developed with the special contribution of the European Heart Rhythm Association (EHRA) of the ESC. Eur. Heart J..

[11] Hart R.G., Pearce L.A., Aguilar M.I. (2007). Meta-analysis: Antithrombotic therapy to prevent stroke in patients who have nonvalvular atrial fibrillation. Ann. Intern. Med..

[12] Ruff C.T., Giugliano R.P., Braunwald E., Hoffman E.B., Deenadayalu N., Ezekowitz M.D., Camm A.J., Weitz J.I., Lewis B.S., Parkhomenko A. (2014). Comparison of the efficacy and safety of new oral anticoagulants with warfarin in patients with atrial fibrillation: A meta-analysis of randomised trials. Lancet.

[13] Potpara T.S., Lip G.Y.H., Blomstrom-Lundqvist C., Boriani G., Van Gelder I.C., Heidbuchel H., Hindricks G., Camm A.J. (2021). The 4S-AF Scheme (Stroke Risk; Symptoms; Severity of Burden; Substrate): A Novel Approach to In-Depth Characterization (Rather than Classification) of Atrial Fibrillation. Thromb. Haemost..

[14] Romiti G.F., Pastori D., Rivera-Caravaca J.M., Ding W.Y., Gue Y.X., Menichelli D., Gumprecht J., Kozieł M., Yang P.-S., Guo Y. (2021). Adherence to the ‘Atrial Fibrillation Better Care’ Pathway in Patients with Atrial Fibrillation: Impact on Clinical Outcomes—A Systematic Review and Meta-Analysis of 285,000 Patients. Thromb. Haemost..

[15] Fitzmaurice D.A., Hobbs F.D.R., Jowett S., Mant J., Murray E.T., Holder R., Raftery J.P., Bryan S., Davies M., Lip G.Y.H. (2007). Screening versus routine practice in detection of atrial fibrillation in patients aged 65 or over: Cluster randomised controlled trial. BMJ.

[16] Dilaveris P.E., Kennedy H.L. (2017). Silent atrial fibrillation: Epidemiology, diagnosis, and clinical impact. Clin. Cardiol..

[17] Freedman B., Camm J., Calkins H., Healey J.S., Rosenqvist M., Wang J., Albert C., Anderson C.S., Antoniou S., Benjamin E.J. (2017). Screening for Atrial Fibrillation: A Report of the AF-SCREEN International Collaboration. Circulation.

[18] Hobbs F.D., Fitzmaurice D.A., Mant J., Murray E., Jowett S., Bryan S., Raftery J., Davies M., Lip G. (2005). A randomised controlled trial and cost-effectiveness study of systematic screening (targeted and total population screening) versus routine practice for the detection of atrial fibrillation in people aged 65 and over. The SAFE study. Health Technol. Assess..

[19] Department of Health and Social Care (2008). Pharmacy in England: Building on Strengths—Delivering the Future. https://www.gov.uk/government/publications/pharmacy-in-england-building-on-strengths-delivering-the-future.

[20] Lowres N., Neubeck L., Salkeld G., Krass I., McLachlan A.J., Redfern J., Bennett A.A., Briffa T., Bauman A., Martinez C. (2014). Feasibility and cost-effectiveness of stroke prevention through community screening for atrial fibrillation using iPhone ECG in pharmacies. The SEARCH-AF study. Thromb. Haemost..

[21] Willis A., Rivers P., Gray L.J., Davies M., Khunti K. (2014). The effectiveness of screening for diabetes and cardiovascular disease risk factors in a community pharmacy setting. PLoS ONE.

[22] Lowres N., Krass I., Neubeck L., Redfern J., McLachlan A.J., Bennett A.A., Freedman B. (2015). Atrial fibrillation screening in pharmacies using an iPhone ECG: A qualitative review of implementation. Int. J. Clin. Pharm..

[23] Statista (2021). Average Number of Morrisons Customers per Store per Week in the United Kingdom from Financial Year 2009/2019 to 2020/2021. https://www.statista.com/statistics/382353/morrisons-weekly-customer-numbers-united-kingdom-uk/.

[24] Public Health England (2015). Atrial Fibrillation Prevalence Estimates for Local Populations. https://www.gov.uk/government/publications/atrial-fibrillation-prevalence-estimates-for-local-populations.

[25] Public Health England (2021). Public Health Profiles. https://fingertips.phe.org.uk/search/atrial#page/0/gid/1/pat/46/par/E39000026/ati/154/are/E38000056.

[26] Carpenter J.M., Moore M. (2006). Consumer demographics, store attributes, and retail format choice in the US grocery market. Int. J. Retail Distrib. Manag..

[27] Lowers N., Neubeck L., Redfern J., Freedman S. (2013). Screening to identify unknown atrial fibrillation. Thromb Haemost..

[28] Vaes B., Stalpaert S., Tavernier K., Thaels B., Lapeire D., Mullens W., Degryse J. (2014). The diagnostic accuracy of the MyDiagnostick to detect atrial fibrillation in primary care. BMC Fam. Pract..

[29] Tieleman R.G., Plantinga Y., Rinkes D., Bartels G.L., Posma J.L., Cator R., Hofman C., Houben R. (2014). Validation and clinical use of a novel diagnostic device for screening of atrial fibrillation. Europace.

[30] Morgan S., Mant D. (2002). Randomised trial of two approaches to screening for atrial fibrillation in UK general practice. Br. J. Gen. Pract..

[31] Dougherty L., Lister S., West-Oram A. (2015). The Royal Marsden Manual of Clinical Nursing Procedures.

[32] Braun V., Clarke V. (2006). Using thematic analysis in psychology. Qual. Res. Psychol..

[33] Boriani G., Laroche C., Diemberger I., Fantecchi E., Popescu M.I., Rasmussen L.H., Sinagra G., Petrescu L., Tavazzi L., Maggioni A.P. (2015). Asymptomatic atrial fibrillation: Clinical correlates, management, and outcomes in the EORP-AF Pilot General Registry. Am. J. Med..

[34] Potpara T.S., Polovina M.M., Marinkovic J.M., Lip G.Y. (2013). A comparison of clinical characteristics and long-term prognosis in asymptomatic and symptomatic patients with first-diagnosed atrial fibrillation: The Belgrade Atrial Fibrillation Study. Int. J. Cardiol..

[35] Siontis K.C., Gersh B.J., Killian J.M., Noseworthy P.A., McCabe P., Weston S.A., Roger V.L., Chamberlain A.M. (2016). Typical, atypical, and asymptomatic presentations of new-onset atrial fibrillation in the community: Characteristics and prognostic implications. Heart Rhythm.

[36] Steinhubl S.R., Waalen J., Edwards A.M., Ariniello L.M., Mehta R.R., Ebner G.S., Carter C., Baca-Motes K., Felicione E., Sarich T. (2018). Effect of a Home-Based Wearable Continuous ECG Monitoring Patch on Detection of Undiagnosed Atrial Fibrillation: The mSToPS Randomized Clinical Trial. JAMA.

[37] Welton N.J., McAleenan A., Thom H.H., Davies P., Hollingworth W., Higgins J.P., Okoli G., Sterne J., Feder G., Eaton D. (2017). Screening strategies for atrial fibrillation: A systematic review and cost-effectiveness analysis. Health Technol. Assess..

[38] Nielsen J.C., Lin Y.J., de Oliveira Figueiredo M.J., Sepehri Shamloo A., Alfie A., Boveda S., Dagres N., Di Toro D., Eckhardt L.L., Ellenbogen K. (2020). European Heart Rhythm Association (EHRA)/Heart Rhythm Society (HRS)/Asia Pacific Heart Rhythm Society (APHRS)/Latin American Heart Rhythm Society (LAHRS) expert consensus on risk assessment in cardiac arrhythmias: Use the right tool for the right outcome, in the right population. J. Arrhythmia.

[39] Li K.H.C., White F.A., Tipoe T., Liu T., Wong M.C., Jesuthasan A., Baranchuk A., Tse G., Yan B.P. (2019). The Current State of Mobile Phone Apps for Monitoring Heart Rate, Heart Rate Variability, and Atrial Fibrillation: Narrative Review. JMIR Mhealth Uhealth.

